# Primary healthcare professionals’ perspectives on patient help-seeking for lung cancer warning signs and symptoms: a qualitative study

**DOI:** 10.1186/s12875-022-01730-x

**Published:** 2022-05-18

**Authors:** Mohamad M. Saab, Michelle O’Driscoll, Serena FitzGerald, Laura J. Sahm, Patricia Leahy-Warren, Brendan Noonan, Caroline Kilty, Noreen Lyons, Heather E. Burns, Una Kennedy, Áine Lyng, Josephine Hegarty

**Affiliations:** 1 Catherine McAuley School of Nursing and Midwifery, University College Cork, College Road, T12 AK54 Cork, Ireland; 2 School of Pharmacy, University College Cork, Cork, Ireland; 3 Rapid Access Lung Clinic, Cork University Hospital, Cork, Ireland; 4 National Cancer Control Programme, Health Service Executive, Dublin, Ireland

**Keywords:** Early detection, Focus group, General practitioners, Help-seeking behavior, Lung cancer, Nurses, Pharmacists, Primary care, Qualitative research

## Abstract

**Background:**

Lung cancer is the leading cause of cancer incidence and mortality worldwide. Prompt patient help-seeking for signs and symptoms suggestive of lung cancer is crucial for early referral, diagnosis, and survivorship. However, individuals with potential lung cancer symptoms tend to delay help-seeking. This qualitative study explored perceived barriers to patient help-seeking and strategies to enhance help-seeking for lung cancer warning signs and symptoms from the perspective of primary healthcare professionals.

**Methods:**

Semi-structured focus groups and individual interviews were conducted with 36 primary healthcare professionals. Data were collected via videoconferencing. Inductive thematic analysis was conducted.

**Results:**

The following two themes were created from the data: (i) perceived barriers to patient help-seeking for signs and symptoms of concern and (ii) facilitating early patient presentation for signs and symptoms of concern. Some participants believed that the high cost of a general practitioner visit, long waiting times, and previous bad experiences with the healthcare system would deter patients from seeking help for symptoms of lung cancer. Perceived patient-related barriers to help-seeking related to the different emotions associated with a potential cancer diagnosis as well as stigma, embarrassment, and guilt felt by smokers. Sociodemographic factors such as drug use, homelessness, living in rural areas, and being male and older were also perceived to impede patient help-seeking. The negative impact of the COVID-19 pandemic on cancer help-seeking also featured strongly. Participants recommended several strategies to enable patients to seek help for symptoms of concern including targeted educational campaigns focussing on symptoms (e.g., cough) rather than behaviours (e.g., smoking), accessible and free health services, and using patients’ support networks.

**Conclusions:**

Patient-related and healthcare system-related barriers to help-seeking for lung cancer warning signs and symptoms include cost of healthcare, cancer fear, and various sociodemographic factors. Participants suggested that increased awareness and early patient help-seeking for symptoms of concern could be achieved through targeted patient education, national campaigns, the use of community support networks, and free and accessible targeted screening services.

## Background

With an estimated 2.2 million new cases and 1.8 million deaths in 2020, lung cancer (LC) remains the leading cause of cancer incidence and mortality in men and women worldwide [1]. By 2040, these figures are projected to increase to 3.63 million new cases and 3.01 million deaths [2]. In the Republic of Ireland, LC is the fourth most diagnosed invasive cancer with 1503 men and 1250 women diagnosed each year. However, LC remains the leading cause of cancer mortality, with 1039 men and 845 women dying from it annually [3]. LC incidence is projected to increase by 131% for males and 105% for females by 2045 in the Republic of Ireland [4].

Early signs and symptoms of LC include a new cough, changes to an existing cough, and shortness of breath [5]. Early stage LC can also be asymptomatic. Haemoptysis is strongly associated with advanced LC but occurs in only a fifth of patients diagnosed with LC [6]. Other signs of late-stage disease include weight loss and fatigue [7]. Therefore, the symptom signature of LC is broad [8], which may contribute to delay in patient presentation and subsequent LC diagnosis.

Early patient help-seeking for signs and symptoms suggestive of LC is crucial for timely specialist referral and diagnosis. However, patients diagnosed with LC may experience significant delay between symptom onset and initiation of treatment for various reasons, including patient factors, healthcare professional factors, and healthcare system factors [5, 8]. For example, one study found that LC stigma impacts negatively on help-seeking, leading to a median waiting time of 41 days from symptom onset to medical help-seeking [9]. This delay, coupled with the impact of social and economic inequalities often associated with increased risk of LC [10], has detrimental effects on timely diagnosis, quality of life, cost of healthcare, and patients’ eligibility for curative treatment [6]. In recent years, COVID-19 has also had a detrimental impact on cancer help-seeking and diagnosis [11]. A survey of 7543 adults in the UK found that approximately half of patients who reported experiencing cancer alarm signs and symptoms did not contact their general practitioner (GP) due to concerns about catching or transmitting COVID-19 [11]. Another survey of 3269 individuals in Spain found that participants reported significantly longer waiting times to help-seeking for cancer symptoms due to fears of overburdening the healthcare system during the pandemic [12].

In addition to patient factors such as lack of symptom awareness and symptom misappraisal [13], healthcare professional and healthcare system factors that can contribute to delayed LC diagnosis include poor patient-healthcare professional relationships, lack of healthcare access, and cost of primary care [8, 13]. For instance, in the Republic of Ireland, private patients typically pay between €45 and €65 to see their GP [14], representing a potential financial barrier to accessing primary care. However, adults over 70 and children under six are entitled to a GP visit card, which allows them to visit a GP free of charge. Also, based on individual circumstances, some patients are eligible for a medical card, which enables them to access certain health services, including GPs, for free [14]. Over 30% of people in Ireland are medical card holders [15].

In Ireland, LC is diagnosed through three main routes – Rapid Access Lung Clinics, other outpatient services, or emergently. Rapid Access Lung Clinics aim to provide prompt diagnostic evaluation of patients with clinical/radiological findings suspicious for LC within 2 weeks of referral [16]. Most Rapid Access Lung Clinics referrals originate from GPs [17, 18]. Other primary healthcare professionals, such as practice nurses (PNs), community pharmacists (CPs), and public health nurses (PHNs), cannot refer patients directly to the Rapid Access Lung Clinics. However, they play an important role in the early diagnosis of LC by encouraging patients with symptoms of concern to promptly visit their GP.

While recent studies have explored LC help-seeking from the perspective of at-risk populations [13, 19, 20], there has been very little research conducted amongst primary healthcare professionals who play a key role in advising and referring patients who present to them with signs and symptoms suggestive of LC, particularly during the COVID-19 pandemic. Therefore, this study explored perceived barriers to patient help-seeking and strategies to enhance help-seeking for LC signs and symptoms from the perspective of primary healthcare professionals in Ireland.

## Methods

### Research design and paradigm

A qualitative descriptive design was used [21]. Drawing from the naturalistic inquiry, qualitative description is the least theoretical qualitative design since it describes the phenomenon of interest in its natural state rather than adhering to pre-existing theories pertaining to this phenomenon. Moreover, qualitative description is well suited to answer questions and provide recommendations that are pertinent to practitioners and policymakers [15]. The 21-item Standards for Reporting Qualitative Research checklist was used to report this study and maintain an audit trail [22].

### Researcher characteristics and reflexivity

Qualitative interviews and focus groups were conducted by four doctorally prepared academics (MMS, MO’D, LJS, CK) who have a health background and have extensive expertise in qualitative research. Field notes were taken by three other researchers (HEB, UK, ÁL). Researchers were not known to participants. Individual interviews and focus groups were facilitated by two researchers. The first interviewer led the discussion and the second interviewer probed participants and helped ensure that all focus group participants had an opportunity to contribute to the discussion.

Interviewers and field note takers convened directly after each individual interview and focus group and audio-recorded their own reflections on the interview/focus group process. The lead interviewer (MMS) then prepared and shared with the team a summary capturing the essence of the discussions. This process of memoing is known to enhance researcher reflexivity and qualitative data confirmability [23]. It also enables researchers to identify key themes and specify areas that warrant further exploration in subsequent interviews and focus groups [13, 19].

### Sample and context

Non-probability purposive sampling was used to recruit primary healthcare professionals (i.e., GPs, PHNs, PNs, and CPs). Snowball sampling was also used whereby participants were asked to refer their colleagues and encourage them to participate in the study. Only primary healthcare professionals working in the Republic of Ireland were considered for inclusion in this study. Healthcare professionals working in other jurisdictions were not eligible for inclusion due to differences in healthcare systems.

Various professional bodies were contacted by e-mail and asked to circulate to their members the study invitation letter, poster, and a link where primary healthcare professionals registered their interest in participating. In addition, CPs were invited to participate during a LC webinar and via e-mail communications from colleagues.

### Data collection methods and instruments

This study received ethical approval from the Social Research Ethics Committee at University College Cork, Ireland on January 15, 2021. Data were collected between February and April 2021. Primary healthcare professionals who were interested in participating and who provided their contact details were contacted by a researcher (MO’D) to arrange for data collection. Participants were provided with a study information leaflet. They were then asked to electronically sign informed consent and complete an eight-item sociodemographic questionnaire containing questions on age, gender, highest level of education, years of experience, current role, time in current role, county of work, and area of work.

Due to the COVID-19 pandemic, all study documents were completed and returned to the researchers electronically, and data were collected via videoconferencing. Participants were invited to participate either in focus groups or in individual interviews to facilitate the participation of primary healthcare professionals who have busy schedules, particularly during the COVID-19 vaccination rollout. Of note, the combination of two or more qualitative data collection methods is known to enhance data richness, depth, and trustworthiness [24].

All individual interviews and focus groups were audio-recorded and guided by a semi-structured topic guide (Table 1). Participants were also invited to comment on posters and leaflets from two National Health Service patient-focussed LC awareness campaigns in England (Be Clear on Cancer; https://www.nhs.uk/be-clear-on-cancer/symptoms/lung-cancer) and Scotland (Get Checked Early; https://getcheckedearly.org/lung-cancer). Each participant received a €20 gift voucher.

**Table 1:** Topic guide for individual interviews and focus groups

1. I would like you to reflect on help-seeking for lung cancer: a. What do you think would stop a person from seeking help from a GP/public health nurse/practice nurse/community pharmacist for symptoms indicative of lung cancer? b. What do you think would encourage a person to seek help from a GP/public health nurse/practice nurse/community pharmacist for symptoms indicative of lung cancer?	
2. Last year, we spoke to several individuals who were at risk for lung cancer. A number of these individuals refused to seek help from their GP due the (i) cost of a GP visit, (ii) previous bad experiences with the healthcare system, (iii) long waiting time to get a GP appointment, and (iv) some GPs’ perceived negative attitudes towards smokers (e.g., shaming and blaming everything on smoking). a. What do you think can be done to address such barriers? b. Some participants indicated that as a first step they would go to their local pharmacy and buy a cough syrup. What are your views on this? *(probes: opportunity to discuss the ‘alarm’ symptoms).*	
3. Share with participants the (i) “Be Clear on Cancer” poster, leaflet and symptom checker card and (ii) “Detect Cancer Early” poster and leaflet, give them 5-10 min to go over those, then ask: a. How did you find the interventions? *(probes: format, colour, information, celebrity, personal stories)* b. Do you think these resources would be useful to your patients? If so, which one(s)? Why?	

### Data processing and analysis

Interviews and focus groups used an encrypted digital audio recorder and were transcribed verbatim by confidential transcription services. All transcripts were pseudonymised and cross-checked for accuracy against the audio recordings by one researcher (MO’D). Audio recordings were then deleted permanently. Electronic consent forms and sociodemographic questionnaires were stored on an encrypted and password protected computer, accessible to the lead (MMS) and postdoctoral (MO’D) researchers. Sociodemographic questionnaires were deleted following data analysis.

Transcribed data were analysed using inductive thematic analysis [25]. Data analysis was iterative and began immediately after the first interview, such that analysis of early interviews informed the content of subsequent interviews. Excerpts from participants were extracted and shortened into codes. A coding sheet was then created with codes in one column and participant experts in a second column. Data from each participant group (i.e., GPs, PHNs, PNs, and CPs) were coded individually by one researcher (MO’D). All codes were cross-checked for accuracy, collapsed, and refined in agreement with the full researcher team. Data source triangulation was then performed by two researchers (MMS, MO’D). This involved collating all the codes and exploring similarities and differences in the data. Sub-themes linking the various codes were generated and cross-checked against participants’ excerpts. Similar sub-themes were then grouped into themes. IBM® SPSS® software platform was used to produce the participant sample characteristics.

### Trustworthiness

Dependability was established by having the research team cross-check the coding process and agree on the analysis. Transferability was enhanced by providing an audit trail and selecting a heterogenous sample of primary healthcare professionals working across 11 Irish counties. The use of participants’ own words to present data helped improve data credibility and the use of icebreakers prior to data collection helped enhance authenticity.

## Results

### Sample characteristics

A total of 36 primary healthcare professionals participated in this study. Focus groups lasted between 56 and 86 minutes and individual interviews lasted between 25 and 55 minutes. The number and duration of individual interviews and focus groups by participant group (i.e., GPs, PHNs, CPs, and PNs) are presented in Table 2.

**Table 2:** Number and duration of individual interviews and focus groups per participant group

Participant groups	Number of participants	Number of individual interviews	Number of focus groups	Number of participants in focus groups	Interview duration (minutes)
General Practitioners	8	4	1	4	212
Public Health Nurses	10	1	3	2, 2, 5	253
Community Pharmacists	10	0	2	5, 5	167
Practice Nurses	8	0	2	2, 6	148
Total	36	5	8	31	780

The majority or participants (80.5%) were female and held either a bachelor’s (30.6%) or master’s degree (30.6%). Years of experience and time in current role ranged widely from 1 to 36 years and 1 to 26 years respectively (Table 3).

**Table 3:** Sample characteristics (*n* = 36)

Sample characteristics		n (%)
**Age range (years)**	21–30	5 (13.9)
	31–40	5 (13.9)
	41–50	13 (36.1)
	51–60	11 (30.6)
	> 60	2 (5.6)
**Gender**	Female	29 (80.5)
	Male	7 (19.5)
**Highest level of education**	Diploma	1 (2.8)
	Higher/postgraduate diploma	6 (8.3)
	Bachelor’s	11 (30.6)
	Master’s	11 (30.6)
	PhD/Doctorate	3 (8.3)
	Other	4 (11.1)
**Years of experience since primary qualification**	Range: 1–36	
	Mean: 21.67 (±10.53)	
**Current professional role**	Pharmacist	10 (27.8)
	Public health nurse	10 (27.8)
	Practice nurse	8 (22)
	General practitioner (qualified)	7 (19.4)
	General practitioner (trainee)	1 (0.3)
**Time in current professional role (years)**	Range: 1–26	
	Mean: 12.3 (±8.8)	
**County of work**	Cork	18 (50)
	Mayo	5 (13.9)
	Dublin	4 (11.1)
	Waterford	2 (5.6)
	Other	7 (19.6)
**Area of work**	Urban	22 (61.1)
	Rural	14 (38.9)

The following two themes were created from the data: (i) perceived barriers to patient help-seeking for signs and symptoms of concern and (ii) facilitating early patient presentation for signs and symptoms of concern (Table 4). The number that appears next to the abbreviations CP, GP, PHN, and PN corresponds to the number of focus group/individual interview. For example, CP1 corresponds to the first focus group conducted with community pharmacists.

**Table 4:** Study themes, sub-themes, codes, and sample participant excerpts

Themes	Sub-themes	Codes	Sources	Sample participant excerpts
Perceived barriers to patient help-seeking for signs and symptoms of concern	Healthcare system-related barriers to help-seeking	• High cost of a GP visit	GP, CP, PHN, PN	*“Where I’m working, we have a huge volume of patients with medical cards, and I’d find that they’re much more ready. They want to talk to you. They want to show you everything and they’re nearly asking you, ‘Should I go to the GP? I think I’ll call the doctor…It’s a different environment or it’s a different ethos”* (CP2)
		• Waiting times to see a GP and time constraint	GP, CP, PHN, PN	*“I’m suffering myself. I’m trying to find a GP. Just for myself with a GP and I’ve asked over 15 practices around…and nothing”* (PN1)
		• Bad experiences with mis/delayed diagnosis, and chest X-ray failure to detect lung cancer	GP, PHN, PN	*“They* [family] *had great faith in their GP and they were very happy with him generally, but they just felt that he was not acting on the fact that she was deteriorating so quick, on how they would like it to proceed. And they felt they had to circumvent him”* (PHN1)
	Patient-related barriers to help-seeking	• Embarrassment, guilt, and fear of judgement due to smoking history	GP, CP, PHN, PN	*“If people are smokers, they feel that perhaps I brought this on myself and I’ve nobody to blame but myself. So, bury their head in the sand”* (PN2)
		• Emotional factors: cancer fear, denial, and anger	GP, CP, PHN, PN	*“Our patients are very busy. Their heads and their lives are very full just running around sorting stuff out…they literally haven’t got the bandwidth for contemplating something major like that* [cancer diagnosis]. *They just don’t want to think about it, so they just act like it’s not happening…then it’s up to us to pick it up…”* (GP5)
		• Sociodemographic factors: educational level, drug use, homelessness, and being male and older	GP, CP, PHN, PN	*“It’s always the wife who rings up, isn’t it?* [laughter] *to the public health nurse centre…especially with the older male, you tend to get a bit of that. So that’s something I would have experienced…”* (PHN4)
	The impact of the COVID-19 pandemic on patient help-seeking	• Stigma and embarrassment relating to cough	CP, PN	*“That’s a huge thing at the moment that I’m recognising. If someone comes in with a cough that’s totally unrelated to COVID, they nearly feel embarrassed. So, I think in this instance, that might be a barrier to presentation as well for people on a practical level”* (CP2)
		• Lack of in-person contact with healthcare professionals	GP, CP, PN	*“I’ve so many patients who are just getting antibiotic prescriptions emailed in and it’s the patients who aren’t going to the GP because they’re supposed to be cocooning and they’re not even getting seen. So, they’re not even having their chest listened to and they’re having antibiotic prescriptions sent over and I’m not even seeing them because there’s a family member coming in to pick it up. So, I think that’s a real barrier”* (CP1)
		• COVID-19-related health issues prioritised	CP, PHN, PN	*“When COVID started, nobody knew where we were and what was happening. Clinics were being cancelled. Patients were ringing us going: ‘This clinic has been cancelled. What do I do now? Where do I go? I need advice on my inhalers.’ Everybody was stuck. I think we’re still a bit stuck”* (PN1)
		• Fear of contracting or transmitting COVID-19 in healthcare settings	CP, PHN, PN	*“Because of the COVID scares in hospital infections, people have definitely put off accessing treatment for any symptoms. It’s not just specific to lung cancer and talking amongst ourselves in the practice, I’m in a primary care centre, we would definitely associate three or four sudden deaths in the past six months, that was really missed care because of COVID where they’ve put off accessing care and it turned out that other things happened, whether it was an MI* [myocardial infarction] *or whatever…”* (PHN1)
Facilitating early patient presentation for signs and symptoms of concern	Recommendations to promote patient help-seeking	• Patient education	GP, PN	*“They* [patients] *may not be fully aware that GPs have a vital access to lung cancer treatment, and I think maybe emphasising that, they probably see lung cancer as being more a hospital-based thing and not seeing the GP as the gateway to that secondary care”* (GP2)
		• Accessibility of additional and free services for LC health checks/health screening and diagnosis	GP, CP, PHN, PN	*“What we did when we started, it is we sent out a message to all of our patients in that category and we offered them a free screening service, FREE in big letters because people like to get things for free”* (PN1)
		• The positive role of family, GP, and community supports	GP, CP, PHN, PN	*“The home helps are quite good. You’ve a lot of people living alone in our area and the only one that they see or may be in contact with that they’d confide in. The family obviously would be in contact, but they maybe God knows where. They’re not on the ground. And I find home helps are brilliant. They would ring up and they’re saying that they are a little bit concerned seemingly…even people that actually deliver the meals on wheels. That sounds probably a little bit mad, but like that’s the only port of contact for a lot of these people living on their own. But they seem to be on the alert a lot…their role is nearly underestimated…”* (PN2)
	Perspectives on previous patient-focussed campaigns	• Risk of information overload in both campaigns	GP, CP, PHN, PN	*“I like the poster* [Be Clear on Cancer]. *I find the leaflets, although I find them interesting and I think medical people would read through it, I think they’re actually quite wordy for people who aren’t medically minded”* (PHN1)
		• Mixed views on the visuals of both campaigns	GP, CP, PHN, PN	*“I liked the green* [Be Clear on Cancer]…*I think green is a colour of calm…”* (PN2) *“It* [Detect Cancer Early leaflet] *draws your attention…eye catching and identifiable”* (GP4)
		• The risks and benefits of using patient, doctor, and celebrity profiles in both campaigns	GP, CP, PHN, PN	*“The NHS England one* [Be Clear on Cancer] *just has sort of random doctors on it. I’ve never heard of them. Can’t really identify with them. This* [Detect Cancer Early] *is more eye catching and it’s got Fergie* [Alex Ferguson] *on it, so immediately you’re going to pay attention and it’s got the symptoms on it. It’s nice. It’s accessible. It’s not overly text driven. It’s a slicker leaflet. It resonates with you better plus just simple things like the photo, it draws your attention. The other one is sort of sterile and you can’t really engage with it on the same level”* (GP4)
		• The benefits of the catchy slogan and strapline of the “Detect Cancer Early” campaign	GP, CP, PHN, PN	*“The take-home message* [Detect Cancer Early] *is catch it earlier and you’ve got a better chance. Don’t get scared, get checked and it doesn’t have to mean game over. It’s not what it used to be. I like that one”* (GP3) *“You’re not wasting anyone’s time* [Be Clear on Cancer]*. That’s a very useful point actually. That’s really good. Your GP wants to see you. There’s lots that can be done to treat lung cancer these days, especially if it’s found early”* (GP1)
		• Practicality and usability of leaflets for patients queried	GP, CP, PHN, PN	*“Leaflet was too long, and that patients would get bogged down in the detail of it”* (GP3) *“The card for the wallet I’d say will be in there and that’s where it’ll stay, to be honest with you* [Laughter]*”* (PHN4)

*CP* Community Pharmacist, *GP* General Practitioner, *PHN* Public Health Nurse, *PN* Practice Nurse

### Perceived barriers to patient help-seeking for signs and symptoms of concern

Participants identified several healthcare system-related factors and patient-related factors that they believed would deter patients from seeking help for LC warning signs and symptoms. The negative impact of the COVID-19 pandemic on help-seeking was also discussed at length.

#### Healthcare system-related barriers to help-seeking

There was a clear divide in responses while comparing LC help-seeking among GP visit/medical card holders and self-payers. For instance, PNs and CPs found it much easier to encourage patients who have GP visit/medical cards to seek help from their GP as a first step to secondary care referral. While they appreciated the cost of a GP visit as an issue, PHNs noted that many of their service users are medical card holders, for whom financial cost should not represent a barrier to accessing primary care (PHN3, PHN4). However, some GPs believed that the cost of a GP visit would not *“come into play”* (GP2) if serious signs and symptoms of LC were present.

Perceived long waiting times for GP appointments or other health services was also identified as a potential barrier to help-seeking. CP2 noted that people who work during the day might not find the time to visit their GP and PN2 referred to long waiting times to secure a GP appointment based on a personal experience. In contrast, GP2 stated *“not buy*[ing] *into the waiting times argument if someone had symptoms that were serious and if they had a background of smoking.”*

Previous negative patient experiences with the healthcare system and stories of misdiagnosis or delayed LC diagnosis were also identified as potential healthcare system-related barriers to patient help-seeking. Participants seemed aware of the potential for missed diagnoses associated with reliance on X-rays as compared to computed tomography (CT) scans and how normal X-ray findings can impact subsequent patient help-seeking. GP4 offered examples of symptoms being misappraised by GPs and PHN1 gave examples of where delayed LC diagnosis resulted from GPs not listening to the family’s concerns about a patient who *“was deteriorating so quick.”* As a result, the family decided to *“circumvent him* [GP]*.”*

#### Patient-related barriers to help-seeking

The possibility of a cancer diagnosis was perceived to trigger *“fear of dying”* (PHN1) and fear of a *“quick and painful”* death among patients (GP1). As a result, participants believed that patients choose to *“bury their heads in the sand”* (PN1) and *“do not want to upset family”* (GP1). A fatalistic attitude of *“what happens, happens”* (CP2) among some patients adds to the complexity of help-seeking. It was also highlighted that many patients at risk of LC are socioeconomically deprived with multiple stressors, and as a result *“are very busy…*[they] *haven’t got the bandwidth for contemplating something major like that* [cancer diagnosis]*”* (GP5).

The stigma associated with smoking was discussed. It was felt that “*smokers blame themselves for their symptoms”* and therefore “*won’t bother the doctor because it’s* [their] *own fault”* (GP5). PN2 spoke of how patients *“recoil”* when asked whether they smoke, and are embarrassed if they do. CPs and PHNs acknowledged that healthcare professionals are prone to lecturing patients about smoking and *“treat*[ing] *them like children”* (PHN3), which could contribute to delayed help-seeking. They recommended that healthcare professionals should remember that *“smoking is a choice”* (CP1).

Participants agreed that sociodemographic and geographical factors play a role in patient help-seeking. For instance, PN1 noted that many patients in rural areas must travel to access services, and often have less well-established transport links. Conversely, PN2 felt that patients in rural areas visited their GPs more often. While PHNs also acknowledged the issue of accessibility, they said that even patients with good geographic access to services may not seek medical help if other barriers were at play, such as fear of cancer or cost of accessing services (PHN3).

Some GPs spoke about the economic deprivation that they experience in their practices. The term *“unworried unwell”* was used by GP5 to describe patients from socioeconomically deprived areas who tend to have multiple co-morbidities at a young age and significant healthcare needs but are less likely to engage with healthcare services due to multiple other stressors in their lives beyond any potential health concerns. Homelessness and drug use were also perceived by GP5 to add to the complexity of help-seeking. Age and gender were also believed to play a role in help-seeking, whereby participants cited a reluctance in older men who had to be prompted by their spouses to seek help: *“it is always the wife who rings up!”* (PHN4), *“women tend to push men to investigate”* (CP1). CP2 believed that men want to *“appear strong”* and stated that may avoid seeking health advice from CPs since pharmacies are *“generally perceived as being more female places rather than male places*.” This was believed to deter older men from *“pluck*[ing] *up the courage to have* [a] *conversation with their pharmacy or pharmacist”* about symptoms of concern.

#### The impact of the COVID-19 pandemic on patient help-seeking

The COVID-19 pandemic presented unique challenges to patient help-seeking. Because cough is a common symptom of COVID-19, people experiencing a new cough were thought by CPs and PNs to be hesitant and at times *“embarrassed”* (CP2) to seek help. Participants also acknowledged the fear that patients had of contracting COVID-19 in healthcare settings (e.g., GP practices, hospitals, and pharmacies), and the resulting reluctance to seek help. PHN1 gave examples of patients delaying care for so long, in other areas of health, that they needed to go *“straight to A&E* [accident and emergency]*”* or situations ending in myocardial infarction or even death.

Lack of face-to-face contact between patients and healthcare professionals throughout the COVID-19 pandemic was another barrier identified by CPs, whose role includes dispensing medication. In the context of the pandemic, CPs described how medications were sometimes prescribed and dispensed without the patient being physically seen by either the GP or the CP. These patients were typically those with multiple health issues who *“are supposed to be cocooning”* (CP1) and who would send a family member to collect the prescription on their behalf. Lack of in-person contact and assessment was also recognised as a significant barrier by GPs and PNs. For instance, GP5 recounted a time that an in-person appointment with a patient for an unrelated issue revealed significant unplanned weight loss that would have gone unmentioned, and therefore unnoticed, during a telephone consultation. Conversely, GP3 recognised the extra accessibility that remote consultations have brought to clinical practice during the pandemic, and their potential long-term benefits in increasing access to primary care.

Participants reported that services were paused or reconfigured, resources were redeployed, and COVID-19 care was prioritised during the pandemic. As a result, patients appeared to perceive COVID-19-related issues as more important than other health issues and were slower to contact their GP about non-COVID-19-related health concerns (PN2). PN1 added that media communications around timely help-seeking for non-COVID-19 health concerns were lacking during the height of the pandemic, adding to the confusion and uncertainly that patients experienced in relation to the services that continued to be available to them during the pandemic.

### Facilitating early patient presentation for signs and symptoms of concern

Participants offered several recommendations to help promote early patient help-seeking for symptoms of concern.

#### Recommendations to promote patient help-seeking

Patient education was perceived to promote early help-seeking. PHN3 believed that “*people don’t know the symptoms or early signs of lung cancer”* and CP1 thought that patient education should steer away from focusing on the underpinning behaviour that increases LC risk (i.e., smoking) and, instead, focus on symptoms of concern (e.g., unresolving cough). Similarly, symptoms to watch out for were flagged by PNs as something that patients lacked knowledge in, particularly in the event of symptom overlap between existing chronic diseases (e.g., chronic obstructive pulmonary diseases) and LC (PN2). Patient education around the potential for improved survival with early diagnosis of LC was also identified as a possible strategy to reduce public misconceptions around LC being inevitably a *“terminal diagnosis”* (GP3). GPs and PNs also stressed the importance of educating patients about the role of the GP in LC referral and the process of further investigation. PNs highlighted that the general population should also know about Rapid Access Lung Clinics.

PHNs spoke about how breast (PHN4), cervical, and oesophageal cancers (PHN3) are *“high on the agenda”* in contrast to LC, due to greater media coverage. Participants felt that learnings from previous successful health awareness campaigns such as the F.A.S.T. (Facial weakness, Arm weakness, Speech problems, and Time to call 112 or 999 for an ambulance) stroke awareness campaign can be used to inform future LC awareness campaigns by using clear, concise, visual, and targeted messages.

The informal role of the patient’s family and support networks in encouraging patients to seek help was evident, with GP1 describing how some patients had been pressurised to present due to concerns from family members. PHN4 described how patients would be more likely to *“follow through”* with the help-seeking process if family support was present. PHN4 also expanded the concept of support and relationships to include relationships with healthcare professionals, claiming that *“relationship is fundamental* [in help-seeking], *whether that’s the relationship with the GP, the relationship with the public health nurse, the relationship within families.”*

PNs acknowledged the role of community supports for patients, particularly those who live alone, by recognising the crucial yet informal role of *“Home Help* [government service aimed to support older people to remain in their own homes for as long as possible and to support informal carers]*”* and *“Meals on Wheels* [organisation that delivers meals to seniors, disabled, and homebound individuals for free or at low cost]*”* (PN2) personnel in noting deteriorations in patients’ health. While no concrete examples were offered on how ‘Home Help’ and ‘Meals on Wheels’ can promote patient help-seeking in the future, PN2 offered anecdotal reports of incidences where ‘Home Help’ and ‘Meals on Wheels’ *“would ring up* [the PN in the GP’s clinic] *and they’re saying that they are a little bit concerned seemingly…that sounds probably a little bit mad, but that’s the only port of contact for a lot of these people living on their own. But they seem to be on the ball, they’re on the alert a lot…their role is nearly underestimated as well at times.”*

It was suggested that the rapid implementation of an effective COVID-19 testing service has come with lots of learnings, and a model upon which to base other health initiatives (GP1). Several participants recommended free and accessible lung health check/ health screening services for smokers to raise the public profile of LC (GP5), as well as universal access to CT scans. Similarly, free GP care was suggested by PHN4, while access to free-of-charge lung CT scans for all and more lung function tests were suggested by PN1.

#### Perspectives on previous lung cancer awareness campaigns

In general, participants preferred the Scottish campaign, describing it as *“less shouty”* (GP5), except for some CPs who felt that while all *“the important information”* (CP2) was being provided by the Scottish campaign, its delivery was not strong enough, and *“was lost”* (CP1). They felt that signposting to CPs as accessible healthcare professionals to consult with initial concerns was omitted, and this was something that should be addressed (CP1). Participants liked the emphasis placed on the message that GPs *“want to see you”* (GP1) in the English campaign, as well as the outlining of important symptoms (GP2) and the positive emphasis that is placed on what can be done to help (CP1). It was felt that the importance of early detection was clearly presented (CP2). Some GPs recommended including additional information on the red flags for LC in the English campaign and cough bottle use in the Scottish campaign (GP3).

In terms of visuals, some participants felt that the English campaign lacked imagery (CP2), although it was impactful as a poster campaign (CP2), as it was *“big and clear*” (PHN1). The leaflet could have been made more dramatic through the appropriate use of images or other visuals (GP3). Some PHNs liked the *“green colour”* as it felt *“calm”* and was *“legible,”* while others commented that it was *“not very vibrant,”* and the use of *“brighter colours and pictures”* would have been welcomed (PN1). The Scottish campaign, on the other hand, was thought to *“draw your attention…eye catching and identifiable”* (GP4) and was more professional in its approach, looking almost like a magazine and not like other leaflets (PHN1). CP1, however, felt that the imagery of tea and toast in the leaflet was a reminder of hospital and did not find it appropriate.

As for the use of doctors and celebrities, some GPs felt that doctors in the English campaign were *“cross* [angry] *looking”* (GP5) and the use of doctors was perceived as *“sterile,”* while others felt that doctors seemed *“benign”* (CP1) and one of them was a well-known TV doctor (GP1). The use of doctors and older white patients was felt to be a limitation and gender balance was a concern in the doctor representation (PHN4). Some participants, however, favoured the attempt of the English campaign to portray doctors as accessible and human and to use real and relatable patient stories (CP1). It was felt that the use of a celebrity (Sir Alex Ferguson) in the Scottish campaign was a worthwhile approach and would generate conversation (PHN4) and encourage patients to read the leaflet and take on board its message (PN1). Sir Ferguson was recognised by most if not all participants and was perceived as somebody who would draw people in, a friendly face, somebody *“trustworthy”* (GP4) and *“relatable,”* particularly for older men (CP2). CPs, however, felt that using a celebrity ran the risk of the person not being recognised and could potentially lessen the campaign’s credibility. PHN1 also noted that the celebrity may not be liked by some and using them can be *“divisive.”*

There were mixed views about the campaign slogans and straplines. “Be Clear on Cancer” was thought to be *“soft and gentle”* by some (GP3), while others found it *“catchy”* and *“clear”* (PHN4). In the Scottish campaign, the use of the phrase “lung cancer doesn’t have to mean game over” was welcomed, as it implied that early detection could have a positive outcome (GP3) and a LC diagnosis was not necessarily a death sentence (CP1). However, for some, the terminology “extra time” and “game over” sounded *“fatalistic”* (GP1, PHN3), suggesting that *“you’ll still die quickly”* (PN1). In keeping with the Scottish campaign, the strapline “don’t get scared get checked” was described as *“snappy”* and *“very good”* by some (CP2, PN2), as it recognised how people may be feeling (PHN3). However, PHN1 did not favour the use of the word *“scared.”*

The practicality of leaflets was queried widely. The six-page leaflet of the English campaign was considered too long, particularly for patients who are *“not medically minded”* (PHN1) due to the risk of *“information overload”* (PN1), with the one-page poster providing a better amount of information than the leaflet. Some PNs and PHNs also felt that patients would get scared reading the leaflet and would not seek medical help as a result. This contrasted with their view of the Scottish campaign, which they felt also had too much text in the four-page leaflet, but was *“less clinical”* (PHN3), contained less jargon (PN2) and was more *“positive and upbeat”* (PN1), and therefore more appropriate to target patients as it assures them that *“lung cancer is not the death sentence that it used to be”* (PN2). PHN4 felt that while leaflets do not get used, posters get glanced at once and then forgotten, unless they potentially were used on billboards, that *“you could see while stopped in your car.”* Wallet/pocket card versions were perceived by PHN1 as impractical and prone to getting lost.

## Discussion

This study explored perceived barriers for patient help-seeking for signs and symptoms indicative of LC and strategies to enable patients to seek help early. Healthcare system-related barriers to help-seeking included the cost of GP visits and long waiting times. These findings are well documented in previous research with at-risk individuals [13, 19, 20]. However, in the current study, there was no full agreement on how cost impacted patients with LC symptoms due to the eligibility of most high-risk groups (i.e., socioeconomically deprived cohorts and patients above the age of 70) for GP visit/medical cards.

Regarding healthcare system-related barriers, study participants acknowledged that perceived negative judgment by primary healthcare professionals prevents some patients from seeking their help for symptoms of concern. This is a common phenomenon in the health literature, where one negative experience with healthcare professionals can undermine future engagement. This barrier is often more pronounced for illnesses that are associated with lifestyle behaviours like smoking [13]. Previous misdiagnosis and mistrust in the healthcare system are also recognised as healthcare system-related impediments to subsequent help-seeking [13, 26]. Such impediments were mentioned by current study participants and are well documented in the wider literature. Our previous research with high-risk individuals in Ireland found that stories of misdiagnosis caused mistrust in the healthcare system among some participants, which led them to delay help-seeking for symptoms of LC [13]. Additionally, a recent systematic review of 64 studies on psychosocial factors influencing cancer help-seeking found that mistrust in medicine deterred patients from seeking medical help for cancer, particularly in low-income and lower middle-income countries [27].

Accuracy of diagnostic imaging was discussed among participants as another potential health system-level barrier to help-seeking. International evidence demonstrates that the probability of missing a LC on CT scan is lower than on chest X-ray [28]. The risk/benefit ratio of diagnostic modalities/pathways requires careful consideration. Potential risks associated with CT scanning include radiation exposure and overdiagnosis. Additionally, the cost of CT scanning far exceeds that of X-ray. However, these risks may be considered acceptable in light of the contribution to substantial LC mortality reduction that early detection via low dose CT scanning can potentially deliver in target high-risk populations [29].

Patient-related factors associated with delayed help-seeking included the fear, denial, and anger that a potential cancer diagnosis can trigger [13, 20]. Such barriers were perceived to disproportionately impact certain sociodemographic cohorts, including males and/or people who are socioeconomically deprived. Participants believed, for example, that pharmacies were more female-friendly environments which could potentially deter men from entering a pharmacy and seeking help from a CP. Therefore, healthcare initiatives directed at increasing men’s engagement with healthcare services must consider factors that are important to men, such as social networks and local gender norms [30]. Socioeconomic deprivation was perceived as another factor influencing LC help-seeking and diagnosis. Indeed, regional differences in LC incidence exist in Ireland, with a trend of increasing LC incidence with increasing deprivation [31]. For instance, age-adjusted rates of LC were approximately 60% higher for the ‘most’ compared with the ‘least’ deprived population quintiles [31].

Participants believed that the advent of the COVID-19 pandemic has exacerbated delays in help-seeking and subsequent LC diagnosis due to fear of contracting COVID-19 in healthcare facilities, stigma surrounding a cough during the pandemic, and prioritisation of COVID-19-related health concerns. This was demonstrated by a survey in the UK which found that approximately half of patients who reported experiencing cancer alarm signs and symptoms did not contact their GP due to concerns about catching or transmitting COVID-19, wasting the GP’s time, or putting an additional strain on healthcare services [11]. While some GPs perceived telephone consultations as helpful during the COVID-19 pandemic, other healthcare professionals felt the lack of in-person contact could lead to missed LC diagnosis, particularly when physical changes such as unplanned weight loss cannot be readily appraised during telephone consultations. CPs also discussed the dangers of remote prescribing without seeing and assessing the patient. While virtual consultations provide an appropriate alternative to in-person consultations in certain circumstances, several challenges exist, including limited staff training in telephone consultations, suboptimal patient-physician interaction, insufficient technical support, concerns around privacy and confidentiality, and inconsistencies in documentation [32].

Participants recommended several approaches in order to improve patient help-seeking for LC warning signs and symptoms. Patient education was highlighted as key to early LC referral and diagnosis, particularly among PHNs who emphasised the importance of educating high-risk patients (e.g., smokers) about LC before symptoms occur. This finding aligns with the PHN role, which includes a focus on health promotion [33]. CPs cited focusing on LC symptoms rather than smoking as another potential strategy to raise patients’ awareness of LC and engage them in early detection and referral. This is crucial, as focusing on smoking cessation rather than LC awareness may trigger feelings of guilt and embarrassment among at-risk individuals, thus deterring them from seeking help for symptoms of concern [13].

Participants recommended using learnings from previous health awareness campaigns to raise the profile of LC and highlight the importance of seeking timely medical attention for LC alarm symptoms. One example cited in several interviews and focus groups was the F.A.S.T. stroke awareness campaign. This media-based campaign was broadcast in Ireland between May 2010 and June 2011 through national television and radio advertising [34]. An interrupted time series study found a significant change in emergency department attendance among patients with reported stroke symptoms and better health outcomes likely associated with this campaign. The long-term effect of this campaign, however, was not sustained [34]. Of note, stroke is a medical emergency necessitating immediate presentation to the emergency department. On the other hand, LC does not typically present as an emergency. However, there are potentially important lessons to be learned from the design and delivery of the F.A.S.T. campaign in terms of how to effectively deliver information to target audiences.

In keeping with patient education and strategies to improve LC help-seeking, a recent systematic review of interventions promoting LC awareness and help-seeking found that the National Health Service campaign ‘Be Clear on Cancer’ was instrumental in increasing help-seeking for LC symptoms and reducing barriers to help-seeking [35–38]. Indeed, help-seeking for cough increased by 63% during the campaign and by 46% 8 weeks later (*p* < 0.001) [36]. The campaign also yielded a 63% increase in GP attendances for symptoms of concern [37]. As for symptom awareness, ‘Be Clear on Cancer’ significantly increased at-risk individuals’ awareness of cough (*p* < 0.001), breathlessness (*p* = 0.024), haemoptysis (*p* < 0.001), chest pain (*p* = 0.015), and unexplained weight loss (*p* < 0.001) as symptoms of LC [36]. However, subsequent evaluations of this campaign demonstrated that the increase in symptom awareness, presentation, and GP-ordered chest X-rays did not translate into increased urgent suspected cancer referrals or changes in clinical outcomes [39, 40]. Moreover, a recent study of public health messaging and strategies to promote timely LC detection with a sample of 46 individuals with multiple LC risk factors living in high-incidence areas in Ireland found that participants preferred government-led multimodal (e.g., print and broadcast media) national campaigns incorporating public health messages that are “**S**imple, clear, honest; **W**orded positively; **I**ncorporating a shock element; **F**eaturing a celebrity, healthcare professional or cancer survivor; and **T**argeted (acronym: **SWIFT**)” (p. 9) [19].

Participants recommended offering free and accessible lung check/health screening services to help detect LC early in asymptomatic at-risk populations (e.g., smokers/ ex-smokers in defined age range). Low-dose CT is the gold standard screening test in jurisdictions that offer LC screening. LC screening programmes have been shown to reduce LC mortality by up to 20% [41, 42]. However, few countries currently operate population-based LC screening programmes, and the uptake of LC screening in countries like the USA remains low [43]. Ireland does not currently operate a LC screening programme. However, a roadmap for cancer screening in Europe, including LC screening, has been published by Science Advice for Policy by European Academies [44]. This roadmap is still under discussion with European health authorities as part of the recently released European Beating Cancer Plan [45].

## Limitations

Despite ensuring trustworthiness in the conduct and reporting of this study, some limitations are worthy of note. While appropriate in the context of the current study, the use of non-probability sampling increases the risk of self-selection bias and impacts on the transferability of findings. As aforementioned, data were collected via teleconferencing; while this approach was feasible in the context of the COVID-19 pandemic, the human element of qualitative research was lacking. In keeping with COVID-19, data were collected during the peak of vaccination rollout in GP clinics. This served as a challenge to recruiting primary healthcare professionals, especially GPs and PNs.

## Implications

This study was commissioned and funded by the National Cancer Control Programme, a directorate of the Health Service Executive, which is the main provider of health and social care services in the Republic of Ireland. The National Cancer Control Programme works with multiple stakeholders, including health service providers to manage, organise, deliver, and evaluate cancer services in Ireland. Priorities of the National Cancer Control Programme include delivering recommendations of Ireland’s National Cancer Strategy 2017–2026, including recommendation 7 which states that “the NCCP [National Cancer Control Programme] and the HSE [Health Service Executive] Health & Wellbeing Directorate, in partnership with the voluntary sector, will develop a rolling programme of targeted multi-media based public awareness and education campaigns, aimed at the early detection of specific cancers and with particular focus on at-risk populations” (p.134) [46].

Findings from this study and from our earlier study with high-risk individuals [13, 19] will be used by the National Cancer Control Programme to inform the development of initiatives to “push” patients with symptoms of LC to seek help, and to support primary healthcare professionals to “pull” these patients into appropriate services, with the aim of improving cancer outcomes by increasing the proportion of LCs that are diagnosed early in Ireland. Future research is recommended to measure the impact of such interventions on early diagnosis and clinical outcomes, while acknowledging the limitations to inferring a causal relationship between an intervention such an awareness campaign, and an outcome such as a change in the stage distribution of cancers diagnosed.

## Conclusions

**Fig. 1:**
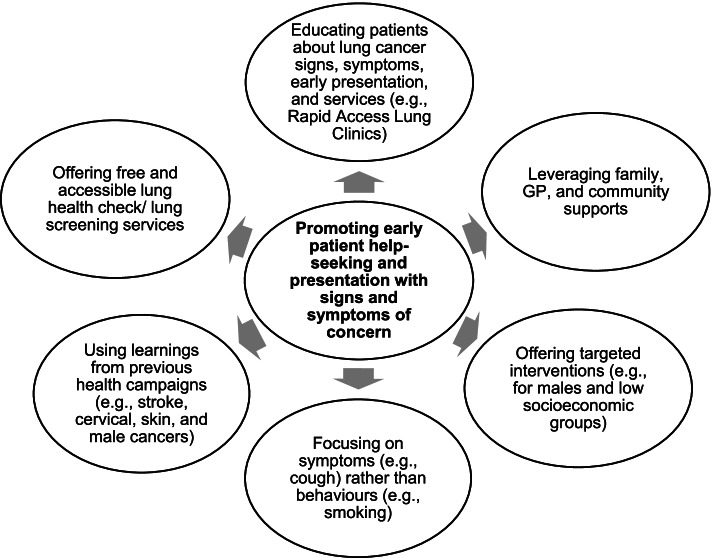
Summary of recommendations to promote early patient help-seeking for symptoms of concern

This study offers rich insights from primary healthcare professionals regarding barriers to patient help-seeking for signs and symptoms suggestive of LC. Several barriers to patient help-seeking were identified such as fear of cancer, embarrassment, sociodemographic factors, the cost of a GP visit, long waiting times, and previous negative experiences with the healthcare system. The COVID-19 pandemic was also perceived to impact negatively on patient help-seeking for respiratory symptoms. Participants recommended several strategies to promote early patient help-seeking such as targeted patient education, using the patient’s support networks, focusing on the symptoms (e.g., unresolving cough) rather than the underlying behaviour (e.g., smoking), using learnings from previous health awareness campaigns, and offering free and accessible lung health checks. These strategies are summarised in Fig. 1.

## Data Availability

All data generated or analysed during this study are included in this published article.

## Abbreviations

CP: Community pharmacist
GP: General practitioner
LC: Lung cancer
PN: Practice nurse
PHN: Public health nurse
